# Feasibility and intra-and interobserver reproducibility of quantitative susceptibility mapping with radiomic features for intracranial dissecting intramural hematomas and atherosclerotic calcifications

**DOI:** 10.1038/s41598-023-30745-2

**Published:** 2023-03-04

**Authors:** Sang Ik Park, Donghyun Kim, Seung Chai Jung, Yoonho Nam, Abdulrahman Alabdulwahhab, Jungbok Lee, Keum Mi Choi

**Affiliations:** 1 Department of Radiology, Chung-Ang University Hospital, Chung-Ang University College of Medicine, 102 Heukseok-ro, Seoul, Republic of Korea; 2 Department of Radiology, Busan Paik Hospital, Inje University College of Medicine, 75, Bokji-ro, Busanjin-gu, Busan, 47392 Republic of Korea; 3 Department of Radiology and Research Institute of Radiology, University of Ulsan College of Medicine, Asan Medical Center, Olympic-ro 33, Seoul, 05505 Republic of Korea; 4 Division of Biomedical Engineering, Hankuk University of Foreign Studies, Yongin-si, Gyeonggi-do Republic of Korea; 5 Radiology Department, Imam Abdulrahman Bin Faisal University, King Fahd Hospital of the University, Dammam, Eastern Province Saudi Arabia; 6 Department of Clinical Epidemiology and Biostatistics, University of Ulsan College of Medicine, Asan Medical Center, Olympic-ro 33, Seoul, 05505 Republic of Korea; 7 Department of Radiology and Research Institute of Radiology, University of Ulsan College of Medicine, Asan Medical Center, 86 Asanbyeongwon-Gil, Songpa-Gu, Seoul, 138-736 Republic of Korea

**Keywords:** Diseases, Neurology

## Abstract

Quantitative susceptibility mapping (QSM) for 61 patients with dissecting intramural hematomas (*n* = 36) or atherosclerotic calcifications (*n* = 25) in intracranial vertebral arteries were collected to assess intra- and interobserver reproducibility in a 3.0-T MR system between January 2015 and December 2017. Two independent observers each segmented regions of interest for lesions twice. The reproducibility was evaluated using intra-class correlation coefficients (ICC) and within-subject coefficients of variation (wCV) for means and concordance correlation coefficients (CCC) and ICC for radiomic features (CCC and ICC > 0.85) were used. Mean QSM values were 0.277 ± 0.092 ppm for dissecting intramural hematomas and − 0.208 ± 0.078 ppm for atherosclerotic calcifications. ICCs and wCVs were 0.885–0.969 and 6.5–13.7% in atherosclerotic calcifications and 0.712–0.865 and 12.4–18.7% in dissecting intramural hematomas, respectively. A total of 9 and 19 reproducible radiomic features were observed in dissecting intramural hematomas and atherosclerotic calcifications, respectively. QSM measurements in dissecting intramural hematomas and atherosclerotic calcifications were feasible and reproducible between intra- and interobserver comparisons, and some reproducible radiomic features were demonstrated.

## Introduction

High-resolution, vessel wall magnetic resonance imaging (VW-MRI) is widely used for the diagnosis of dissection, which is characterized by the presence of an intimal flap, intramural hematoma, double lumen, or aneurysmal dilatation^1,2^. Intramural hematoma, which results from blood entering the subintimal or subadventitial layers^3,4^, shows high signal intensities on a T1-weighted image, and the highest signal intensities are observed in the subacute stage^5^. VW-MRI can also identify atherosclerotic calcifications as a dark signal intensity across all sequences, which is not easy compared to the identification of dissecting intramural hematomas. Susceptibility-weighted imaging (SWI) is a useful processing technique for identifying vessel wall hematoma and calcifications as it presents opposite signal intensity based on phase information^6,7^. Therefore, SWI has been clinically used to qualitatively differentiate intracranial vessel wall hematomas and calcifications.

Quantitative susceptibility mapping (QSM) reconstructed from the phase data of gradient echo provides quantitative information on the local tissue magnetic property by deconvolving the nonlocal field^8,9^. QSM has the ability to differentiate between diamagnetic calcifications and paramagnetic hemorrhage^10–13^ and could thus provide different quantitative values for dissecting intramural hematomas and atherosclerotic calcifications in intracranial vertebral arteries. In addition, quantitative information from QSM could be further used for radiomics, which provides high-dimensional features from imaging data for the construction of diagnostic or prognostic models^14–16^. Given the variable consequences (normalization, steno-occlusion, or dissecting aneurysm) and outcomes of intracranial dissection^17,18^, radiomics-based models might help predict subsequent changes after initiation of intracranial dissection or identify those at risk for developing ischemia or infarction.

Several studies have shown the intra-and/or inter-scanner reproducibility of QSM^19–27^. These studies were focused on the reproducibility of QSM in various structures of the brain parenchyma. Other studies differentiated between hemorrhage and calcification in the brain parenchyma^10^ and extracranial arteries^7^ using QSM. To our knowledge, no studies have evaluated the reproducibility of QSM for dissecting intramural hematomas and atherosclerotic calcifications in the intracranial arteries (including vertebral arteries). Furthermore, the reproducibility of radiomic features is affected by various acquisition and reconstruction methods^15,28–32^, necessitating reproducibility testing prior to building models^33,34^.

The reproducibility of the radiomic features is always questioned due to the nature of the high-dimensional data itself^34^. Therefore, the image biomarker standardization initiative focused on the reproducibility of the radiomic features^35^, and there is a strong association between the reproducibility/repeatability of radiomic features and prognostic values^36^. The lack of reproducibility in radiomic features may easily lead to vulnerable and over-fitted model.

Therefore, this study aimed to evaluate the clinical feasibility and intra- and interobserver reproducibility of QSM and its radiomic features in dissecting intramural hematomas and atherosclerotic calcification in intracranial vertebral arteries.

## Results

### QSM measurements

**Figure 1:**
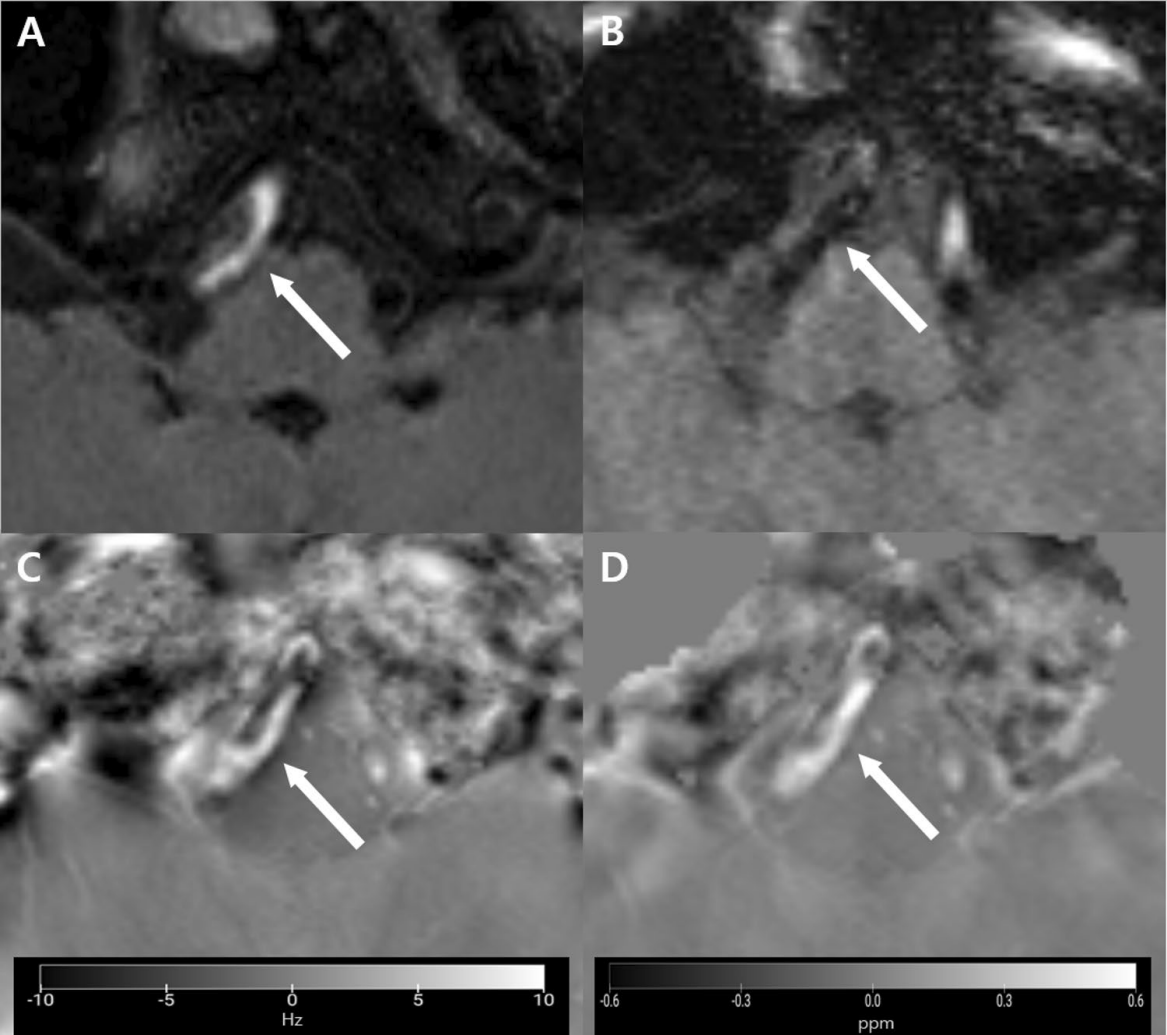
A dissecting intramural hematoma in a 50-year-old male patient. (**A**) An axial precontrast T1-weighted image of vessel wall magnetic resonance imaging (VW-MRI) shows eccentric T1 hyperintensity (arrows) in the wall of the right intracranial vertebral artery, suggesting dissecting intramural hematoma. (**B**) A magnitude image shows dark signal intensity of the lesion (arrows). (**C**) A phase image shows a high signal intensity lesion of the lesion (arrows). (**D**) The lesion has high susceptibility values (mean, 0.328 ppm; median, 0.319 ppm) on quantitative susceptibility mapping (QSM) (arrows), suggesting the paramagnetic nature of the hematoma. The figures were generated using the MRIcroGL version 1.2.20220720 (https://www.nitrc.org/projects/mricrogl/).

**Figure 2:**
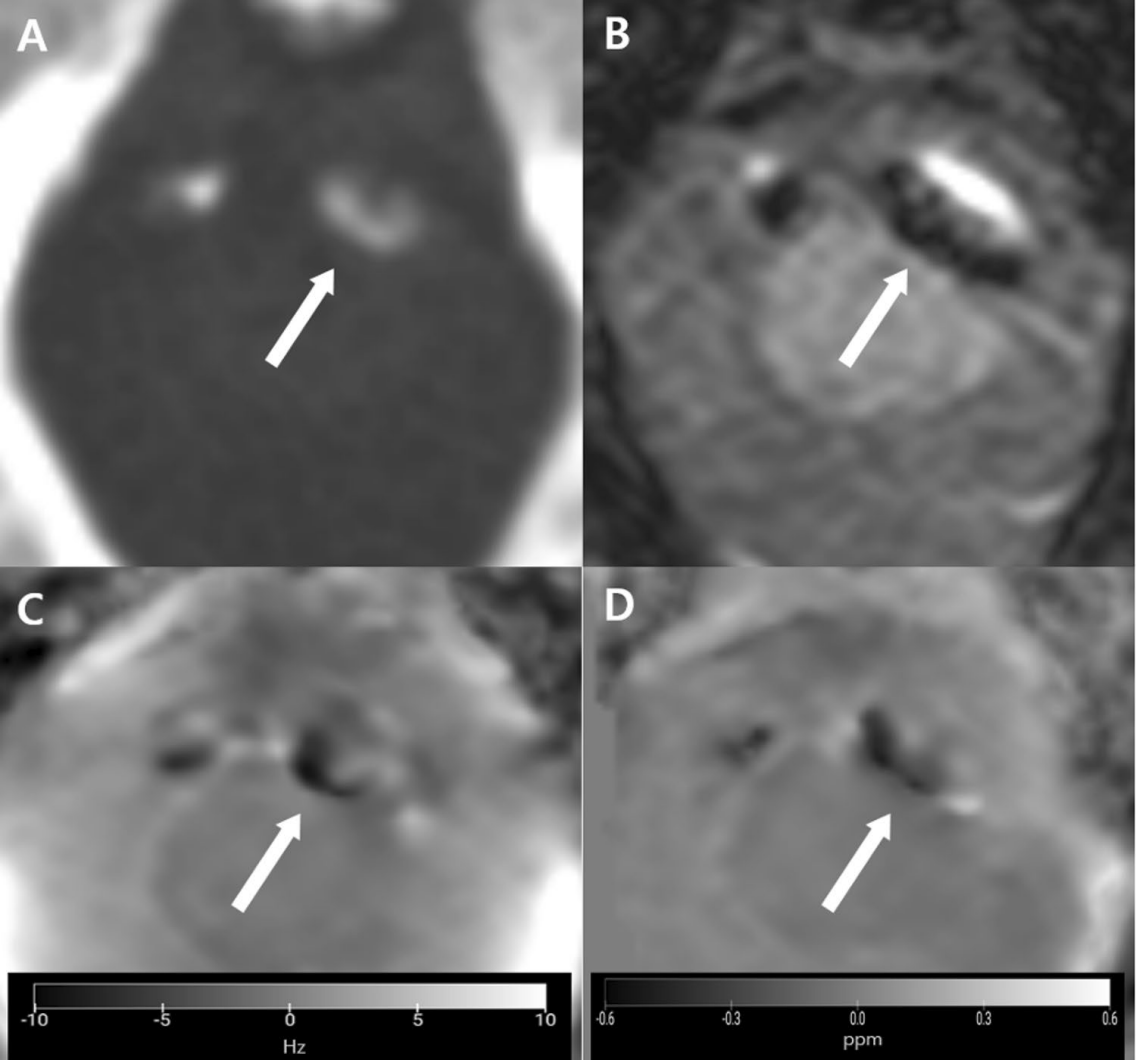
An atherosclerotic calcification in a 68-year-old male patient. (**A**) An axial precontrast CT at the level of the foramen magnum shows wall calcification in the left intracranial vertebral artery (arrow). (**B**) A magnitude image shows a dark signal intensity of the lesion (arrow). (**C**) A phase image shows a heterogeneous low signal intensity lesion of the lesion (arrow). (**D**) The lesions show low susceptibility values (mean − 0.257 ppm; median − 0.265 ppm) on quantitative susceptibility mapping (QSM) (arrow), suggesting the diamagnetic nature of the calcification. The figures were generated using the MRIcroGL version 1.2.20220720 (https://www.nitrc.org/projects/mricrogl/).

Table 1 shows the mean and median QSM values of dissecting intramural hematomas and atherosclerotic calcifications. In all observations, the mean and median QSM values were consistently positive for dissecting intramural hematomas and negative for atherosclerotic calcifications (Figs. 1, 2). Mean and median QSM values were 0.277 ± 0.092 and 0.268 ± 0.094 ppm for dissecting intramural hematomas and -0.208 ± 0.078 ppm and − 0.203 ± 0.078 ppm for atherosclerotic calcifications, respectively.

**Table 1:** QSM values of dissecting intramural hematomas and atherosclerotic calcifications.

Measurement	Dissecting intramural hematoma				Atherosclerotic calcification			
	Observer 1		Observer 2		Observer 1		Observer 2	
	First	Second	First	Second	First	Second	First	Second
Mean (ppm)	0.304 ± 0.095	0.247 ± 0.078	0.297 ± 0.094	0.260 ± 0.092	−0.212 ± 0.078	−0.217 ± 0.080	−0.210 ± 0.073	−0.194 ± 0.083
Median (ppm)	0.287 ± 0.095	0.240 ± 0.078	0.291 ± 0.097	0.254 ± 0.098	−0.207 ± 0.081	−0.210 ± 0.079	−0.206 ± 0.073	−0.188 ± 0.082

*Data indidcate mean ± standard deviation.

### Intra- and interobserver reproducibility in QSM measurements

The intraclass correlation coefficients (ICCs) and within-subject coefficient of variations (wCVs) were 0.885–0.969 and 6.5–13.5% (intraobserver reproducibility), 0.887–0.910 and 11.6–13.7% (interobserver reproducibility), and 0.901–0.915 and 11.1–12.3% (both intra- and interobserver reproducibility) for atherosclerotic calcifications and 0.712–0.785 and 16.1–18.7% (intraobserver reproducibility), 0.823–0.865 and 12.4–14.9% (interobserver reproducibility), and 0.793–0.798 and 15.5–16.0% (both intra- and interobserver reproducibility) for dissecting intramural hematomas. Based on ICCs, reproducibility was excellent in atherosclerotic calcifications and good in dissecting intramural hematomas (Table 2).

**Table 2:** Intra- and interobserver reproducibility of QSM values in dissecting intramural hematomas and atherosclerotic calcifications.

	Dissecting intramural hematoma		Atherosclerotic calcification	
	ICC	wCV (%)	ICC	wCV (%)
Mean				
O1 intra	0.712 (0.003–0.902)	18.7 (14.6–24.2)	0.969 (0.932–0.986)	6.5 (4.8–8.9)
O2 intra	0.785 (0.438–0.906)	16.1 (12.6–20.9)	0.894 (0.755–0.954)	12.7 (9.4–17.6)
1st inter	0.832 (0.697–0.911)	12.9 (10.1–16.7)	0.895 (0.777–0.952)	11.6 (8.6–15.9)
2nd inter	0.865 (0.750–0.929)	12.4 (9.7–16.1)	0.910 (0.637–0.968)	12.1 (8.9–16.8)
Intra and inter	0.793 (0.594–0.895)	15.5 (13.2–18.4)	0.915 (0.846–0.958)	11.1 (9.0–13.9)
Median				
O1 intra	0.746 (0.154–0.905)	17.8 (13.9–23.1)	0.948 (0.887–0.977)	8.7 (6.4–12.0)
O2 intra	0.769 (0.471–0.892)	17.7 (13.8–23.0)	0.885 (0.712–0.951)	13.5 (10.0–18.8)
1st inter	0.823 (0.679–0.906)	13.9 (10.9–18.1)	0.892 (0.770–0.951)	12.3 (9.1–16.9)
2nd inter	0.829 (0.690–0.909)	14.9 (11.6–19.4)	0.887 (0.668–0.956)	13.7 (10.1–19.1)
Intra and inter	0.798 (0.637–0.892)	16.0 (13.6–19.1)	0.901 (0.824–0.950)	12.3 (10.0–15.4)

*ICC* intraclass correlation coefficient, *wCV* within-subject coefficient of variation, *O1* observer 1, *O2* observer 2, *intra* intraobserver reproducibility, *inter* interobserver reproducibility, *1st inter* interobserver reproducibility between the first measurements of observer 1 and 2, *2nd inter* interobserver reproducibility between the second measurements of observer 1 and 2, *Intra* *and inter* reproducibility across the first and second measurements of both observer 1 and 2.

*Parentheses indicate 95% confidence intervals.

**First-order features show both positive and negative values, and thus wCVs were calculated after shifting the values towards either positive or negative sides (towards where a smaller shift was required).

### Intra- and interobserver reproducibility of QSM radiomic features

The number of stable radiomic features (CCC [concordance correlation coefficient] and ICC > 0.85) and median CCCs and ICCs in dissecting intramural hematomas and atherosclerotic calcifications are shown in Table 3. Proportions of reproducible radiomic features across dissecting intramural hematomas and atherosclerotic calcifications were 12% (*n* = 13) in both CCC and ICC (Table 3).

**Table 3:** Intra- and interobserver reproducibility of radiomic features from QSM in dissecting intramural hematomas and atherosclerotic calcifications.

	Dissecting intramural hematoma	Atherosclerotic calcification	Both^a^
Number of radiomic features with CCC > 0.85^b^			
O1 intra	15 (14%)	47 (44%)	13 (12%)
O2 intra	21 (20%)	29 (27%)	11 (10%)
1st inter	19 (18%)	28 (26%)	13 (12%)
2nd inter	16 (15%)	33 (31%)	11 (10%)
Intra and inter	16 (15%)	28 (26%)	13 (12%)
Number of radiomic features with ICC > 0.85^b^			
O1 intra	15 (14%)	47 (44%)	13 (12%)
O2 intra	21 (20%)	31 (29%)	12 (11%)
1st inter	20 (19%)	29 (27%)	13 (12%)
2nd inter	17 (16%)	33 (31%)	12 (11%)
Intra and inter	16 (15%)	28 (26%)	13 (12%)
Median CCC			
O1 intra	0.275	0.792	NA
O2 intra	0.363	0.642	NA
1st inter	0.244	0.575	NA
2nd inter	0.558	0.640	NA
Intra and inter	0.302	0.551	NA
Median ICC			
O1 intra	0.277	0.798	NA
O2 intra	0.368	0.650	NA
1st inter	0.248	0.584	NA
2nd inter	0.563	0.649	NA
Intra and inter	0.306	0.560	NA

*CCC* concordance correlation coefficient, *ICC* intraclass correlation coefficient, *O1* observer 1, *O2* observer 2, *intra* intraobserver reproducibility, *inter* interobserver reproducibility, *1st inter* interobserver reproducibility between the first measurements of observer 1 and 2, *2nd inter* interobserver reproducibility between the second measurements of observer 1 and 2, *Intra and inter* reproducibility across the first and second measurements of both observer 1 and 2.

^a^Number of radiomic features with CCC or ICC > 0.85 in both dissecting intramural hematomas and atherosclerotic calcifications was counted.

^b^Data indicate numbers of reproducible radiomic features and parentheses indicate their proportions.

**Figure 3:**
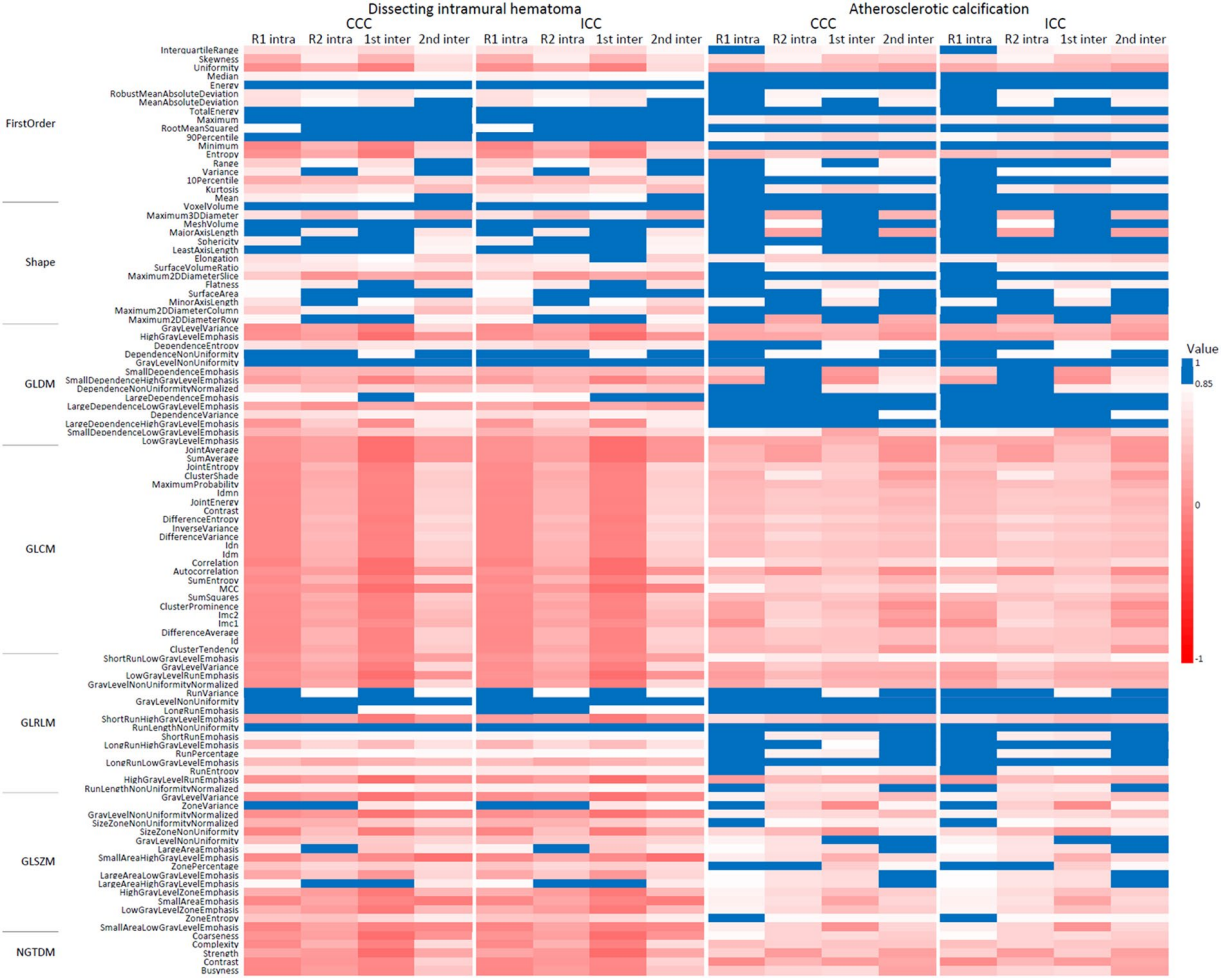
Heatmap of concordance correlation coefficients (CCCs) and intraclass correlation coefficients (ICCs) of individual radiomic features from quantitative susceptibility mapping (QSM) in dissecting intramural hematomas and atherosclerotic calcifications. Blue-colored cells represent stable radiomic features (CCC and ICC > 0.85). *CCC* concordance correlation coefficient, *ICC* intraclass correlation coefficient, *QSM* quantitative susceptibility mapping, *GLDM* gray-level dependence matrix, *GLCM* gray-level co-occurrence matrix, *GLRLM* gray-level run-length matrix, *GLSZM *gray-level size zone matrix, *NGTDM* neighboring gray-tone difference matrix. The heatmap was drawn using the Microsoft Excel version 2211.

There were 9 reproducible features in dissecting intramural hematomas ([1] energy, [2] total energy, [3] maximum, [4] 90th percentile in first-order; [5] voxel volume and [6] mesh volume in shape; [7] gray-level non-uniformity in GLDM; [8] gray-level non-uniformity and [9] run length non-uniformity in GLRLM) and 19 reproducible features in atherosclerotic calcifications ([1] median, [2] energy, [3] total energy, [4] root mean squared, [5] minimum, [6] 10th percentile, [7] mean in first-oder; [8] voxel volume, [9] sophericity, [10] maximum 2D diameter [slice], [11] maximum 2D diameter [column] in shape; [12] gray-level non-uniformity, [13] large dependence emphasis, [14] large dependence low gray-level emphasis, [15] large dependence high gray-level emphasis in GLDM; [16] gray-level non-uniformity, [17] long-run emphasis, [18] run length non-uniformity, [19] long-run low gray-level emphasis in GLRLM) across both CCC and ICC of all observers. Out of the reproducible radiomic features, 6 features ([1] energy, [2] total energy in first-order; [3] voxel volume in shape; [4] gray-level non-uniformity in GLDM; [5] gray-level non-uniformity, [6] run length non-uniformity in GLRLM) were reproducible between dissecting intramural hematomas and atherosclerotic calcification across both observers and both CCCs and ICCs (Fig. 3).

## Discussion

This study demonstrated that QSM measurements were feasible and reproducible in small arteries (such as intracranial vertebral arteries) with representative arterial pathology, dissection, and atherosclerosis. QSM values were positive for dissecting intramural hematomas and negative for atherosclerotic calcifications, which is consistent with paramagnetic hematomas and diamagnetic calcifications, respectively^10^. The reproducibility of QSM values was good to excellent in both dissecting intramural hematomas and atherosclerotic calcifications. A total of 9 and 19 radiomic features (out of 107) were reproducible in dissecting intramural hematomas and atherosclerotic calcifications, respectively.

Some previous studies have evaluated the reproducibility of QSM in vessel walls. Wang et al*.*^37^ showed that QSM and magnetization-prepared rapid acquisition with gradient echo (MPRAGE) had a good agreement and similar sensitivities for the detection of intra-plaque hemorrhage in extracranial carotid arterial atherosclerotic diseases. In this study, Wang et al*.*^37^ presented intra-scanner (scan-rescan) reproducibility of mean susceptibility with only three patients and showed good agreement using Bland–Altman (bias: 0.022 ppm ± 0.101) and liner regression analysis (R^2^ = 0.999; p < 0.0001). Ishii et al*.*^6^ detected microhemorrhages in 24% (12/51) of patients with unruptured intracranial aneurysm using QSM, and all these patients had a history of severe headaches suggestive of sentinel headaches. In the present study, interobserver agreement using kappa for the detection of microhemorrhages in intracranial aneurysm walls was 0.94. To our knowledge, this study is the first to evaluate the intra- and interobserver reproducibility of both quantitative QSM measurements and radiomic features in intracranial arteries. Despite the small size of the lesions, intra- and interobserver reproducibility of the susceptibilities was shown, both in dissecting intramural hematomas and atherosclerotic calcifications. However, 6 out of 107 radiomic features were stable in both dissecting intramural hematomas and atherosclerotic calcifications. The small volume of lesions in the intracranial vertebral arteries might have contributed to the low number of stable features in our study, as previous studies have documented a correlation between stability and volume^38–40^. Among the feature domains, local parameters such as GLCM and NGTDM had no stable features. This is consistent with a previous study on global and local–regional MRI texture features in primary rectal cancer which showed poor repeatability for most local–regional parameters than for global parameters^41^.

Previous studies have demonstrated the feasibility and usefulness of QSM for differentiating calcified and hemorrhagic lesions in locations other than the intracranial vertebral arteries^7,10,37^. Chen et al*.*^10^ evaluated the utility of QSM in distinguishing intracranial calcifications and hemorrhages and reported that QSM has a higher sensitivity and specificity than gradient-recalled echo phase imaging for the detection of intracranial brain parenchymal calcifications and hemorrhages. Ikebe et al*.*^7^ reported higher susceptibility values for intra-plaque hemorrhage and lower susceptibility values for calcifications compared with lipid-rich necrosis in extracranial carotid artery atherosclerotic plaques. Sabotin et al*.*^42^ evaluated 10 fusiform intracranial aneurysms using QSM and reported that 70% (7/10) showed microhemorrhages in the aneurysmal walls. However, they did not try to detect wall calcifications. The quantitative QSM measurements showed higher intra- and interobserver reproducibility in atherosclerotic calcifications than in dissecting intramural hematomas, and radiomic features had higher stability in atherosclerotic calcifications as well. These differences may be attributed to a difference in segmentation. Identification of atherosclerotic calcifications might be easier based on computed tomography angiography (CTA), whereas it might be more difficult to identify dissecting intramural hematomas based on VW-MRI.

QSM may be useful for developing a model using radiomic features even though the differentiation between dissecting intramural hematomas and atherosclerotic calcifications may be easy using mean values. QSM may play a role in predicting the final morphology of intracranial artery dissection given that dissecting intramural hematomas can be altered in terms of amount and shape as the dissection resolves or progresses over time^5^. The extent or amount of atherosclerotic calcification may help predict plaque stability or further stroke risk considering that a higher amount of calcification in atherosclerotic plaques indicates a lower risk of further geometric changes or stroke events^7^. Therefore, investigation of both radiomic features and conventional values for QSM may widen the scope of its usefulness.

The lack of reproducibility of radiomic features may easily lead to a vulnerable and over-fitted model because there is a strong association between reproducibility/repeatability and prognostic values^36^. Intra- and interobserver reproducibility is commonly used during the development of radiomic feature models as a type of reproducibility across algorithms, scanners, and institutes.

There are several limitations to our study. First, this study was retrospective with a small number of patients from a single institution and is thus subject to selection bias. Second, we only measured QSM values in the intracranial vertebral arteries. The intracranial vertebral arteries are the most common location of dissections^43–45^ and the second most common location of atherosclerotic calcifications following the internal carotid artery (ICA), especially the cavernous segment^46,47^. Our study findings cannot be generalized for use in the cavernous segment of the ICA due to severe artifacts adjacent to the skull and air-containing structures related to large field variation^10,37^. Third, reproducibility was tested in a single-site, single-vendor setting, and further studies in multiple sites using MRI machines from different vendors and various protocols are required. Fourth, interobserver reproducibility may be limited by the small number of observers. Fifth, any anisotropic effect associated with vessel orientations may constrain QSM susceptibility^48^. Sixth, the use of ventricles may have potential limitations related to cerebrospinal fluid (CSF) flow or difficulty in drawing the ROIs due to interference by adjacent structures or a small size as the reference region. Seventh, the manual segmentation that we used has a disadvantage even though the vessel wall segmentation has been frequently done based on the manual or semi-automatic method in many studies.

In conclusion, QSM measurements in dissecting intramural hematomas and atherosclerotic calcifications were feasible and reproducible across observations. Moreover, some reproducible radiomic features from QSM values were demonstrated in both dissecting hematomas and atherosclerotic calcifications, which might provide a guide in the choice of features for the future construction of diagnostic or prognostic models.

## Methods

### Patients

**Figure 4:**
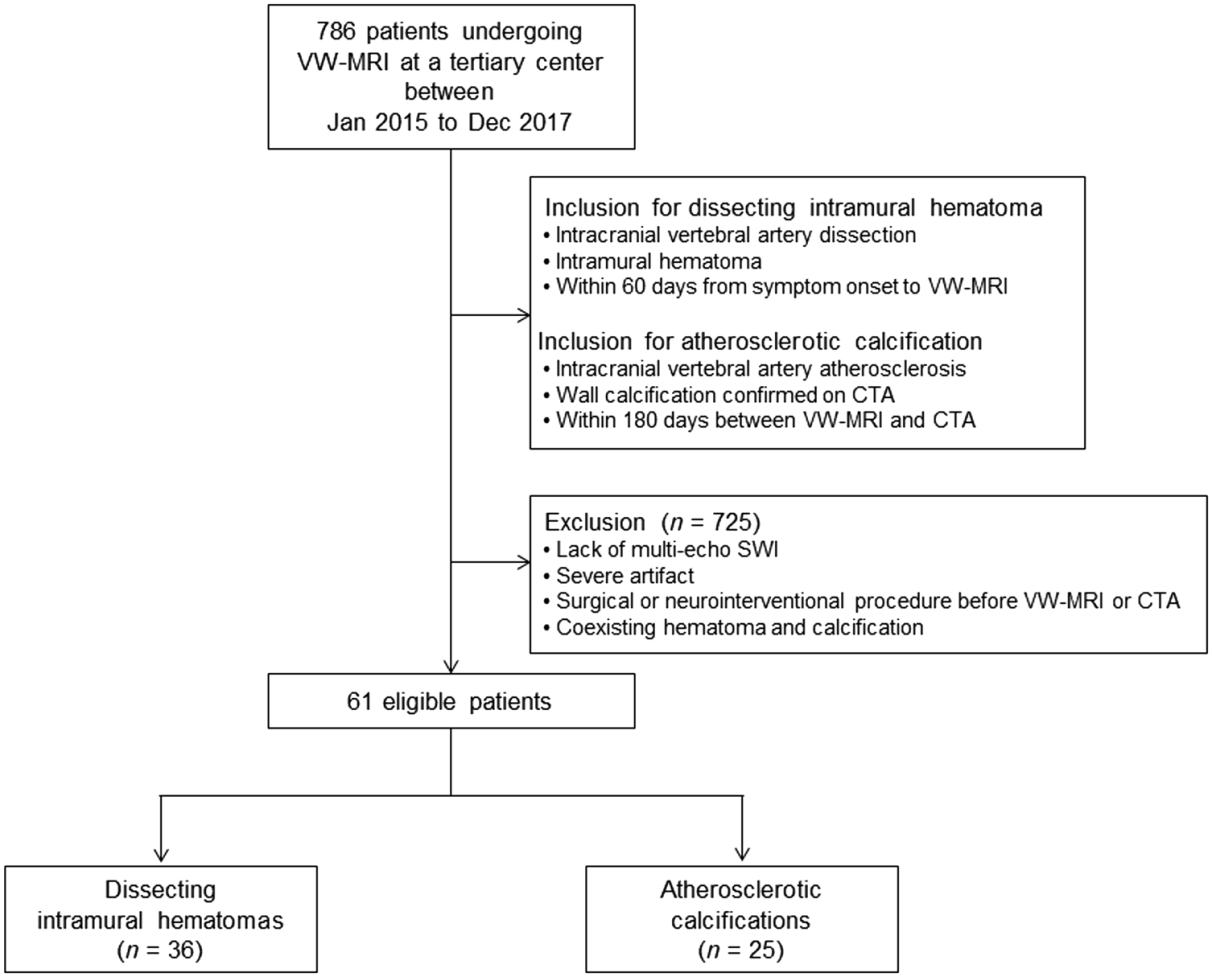
Flowchart of patients.

This retrospective study was approved by the Ethics Committee of Institutional Review Board of Asan Medical Center, Seoul, Republic of Korea, and informed consent was waived. This study follows the international Counsil for Harmonization of Technical Requirements for Registration of Pharmaceutical for Human Use: Guideline for Good Clinical Practice (ICH GCP) and Strengthening the Reporting of Observational Studies in Epidemiology (STROBE) guidelines^49^. Between January 2015 to December 2017, 786 patients underwent VW-MRI for evaluation of intracranial steno-occlusion at a tertiary center. Of them, patients with intracranial vertebral artery dissection having an intramural hematoma of acute to subacute chronology (defined as symptom onset to VW-MRI acquisition ≤ 60 days) were collected for dissecting intramural hematoma. Patients with intracranial vertebral artery atherosclerosis having calcifications (confirmed based on CTA within 180 days of acquisition of VW-MRI) were collected for atherosclerotic calcificiation group. The exclusion criteria for patients with dissecting intramural hematoma were the lack of multi-echo GRE, severe artifacts, and any surgical or neurointerventional procedure for intracranial vertebral artery before the VW-MRI. The exclusion criteria for patients with atherosclerotic calcifications included severe artifacts and any surgical or neurointerventional procedure for intracranial vertebral artery, before or between the VW-MRI and CTA. Patients with a coexisting atherosclerotic calcification and dissecting hematoma in the same segment were also excluded (Fig. 4). The diagnosis of dissecting intramural hematomas or atherosclerotic calcifications was established by the consensus between two neuroradiologists (D.H.K. with 7 years of experience and S.C.J. with 16 years) based on findings of VW-MRI and CTA (detailed diagnostic criteria were described in Supplemental Materials). Sixty-one patients diagnosed with dissecting intramural hematomas (*n* = 36) and atherosclerotic calcifications (*n* = 25) in intracranial vertebral arteries (V4 segments) were selected for this study. Table 4 shows the demographic and clinical characteristics of the 61 patients.

**Table 4:** Demographic and clinical characteristics of patients.

	Dissecting intramural hematoma (*n* = 36)	Atherosclerotic calcification (*n* = 25)	*p* value
Male:female patients	27:9	14:11	0.123
Age, mean (range)	47.4 (19–64)	63.0 (42–86)	< 0.001
Chief complaints, *n* (%)			0.014
Headache	10 (28%)	11 (44%)	
Dizziness	18 (50%)	3 (12%)	
Motor weakness	2 (6%)	3 (12%)	
Asymptomatic	0	3 (12%)	
Others	6 (17%)	5 (20%)	
^a^MRI from symptom, days, mean (range)	16.9 (1–58)	NA	NA
^b^MRI from CTA, days, mean (range)	NA	47.9 (0–171)	NA
Location, *n* (%)			0.021^c^
Unilateral	32 (89%)	16 (64%)	
Right	20 (56%)	8 (32%)	
Left	12 (33%)	8 (32%)	
Bilateral	4 (11%)	9 (36%)	

*MRI* magnetic resonance imaging, *CTA* computed tomography angiography, *NA* not available.

^a^MRI from symptom means time intervals of quantitative susceptibility mapping (QSM) MRI from symptom onset.

^b^MRI from CTA means time intervals between QSM MRI and CTA.

^c^Comparison between unilateral vs. bilateral lesions.

### Imaging protocol

VW-MRI was performed with a 3.0-T MR system (Magnetom Skyra; Siemens, Erlangen, Germany) with a 64-channel head and neck coils. The magnitude and phase images of multi-echo (seven echoes) GRE were obtained for QSM reconstruction, followed by isotropic three-dimensional (3D) MR images. Seven-echo GRE was obtained using a 3D gradient echo sequence (flip angle = 15°; repetition time (TR) = 24 ms; echo times (TE) = 4.92 ms, 7.38 ms, 9.84 ms, 12.30 ms, 14.76 ms, 17.22 ms, and 19.68 ms; matrix size = 320 × 240; field of view = 230 mm × 172 mm; slice thickness = 2 mm; scan coverage   =  140 mm).

3D VW-MRI was performed using sampling perfection with application-optimized contrasts by using different flip-angle evolutions (SPACE), which is a 3D turbo spin-echo sequence^50^. T2-weighted, proton density-weighted, precontrast T1-weighted, and postcontrast T1-weighted images were obtained using SPACE. Postcontrast T1-weighted images were obtained after intravenous administration of gadoterate meglumine (Dotarem; Guerbet, Paris, France) at a dose of 0.1 mmol per kilogram of body weight. The imaging volume ranged from the mandibular angles (approximately proximal internal carotid artery) to the vertex, which allowed for the inclusion of the intracranial vertebral arteries. The acquisition time for each sequence was approximately 8 min. The 3D images were reconstructed into coronal, axial, and sagittal images and displayed with an isovoxel of 0.5 × 0.5 × 0.5 mm^3^. Detailed parameters for VW-MRI were described in Supplemental Materials.

### QSM reconstruction

QSM images were reconstructed from the magnitude and phase images acquired by multi-echo GRE sequence using STI Suite (https://people.eecs.berkeley.edu/~chunlei.liu/software.html) implemented in Matlab R2016a (The Mathworks)^51^. The phase images from the multi-echoes were unwrapped using the Laplacian-based method, then the normalized phase was calculated based on a method by Li et al*.*^52^. The magnitude images were masked to include the intracranial arteries while excluding other noisy regions (clivus and condyles of the occipital bone). The 9 masks were generated by adjusting the fractional intensity threshold using the FSL BET software^53^, and an appropriate mask containing the best ROI including target vessels was selected through visual inspection. Using the normalized phase and the magnitude mask images, the tissue phase images were created after background removal using the magnitude mask and variable-kernel sophisticated harmonic artifact reduction for phase data method. Finally, QSM was calculated using the streak artifact reduction for QSM method^8,9,54,55^. Overall processing of QSM reconstruction took about 5 min for each patient on a personal computer equipped with a 1.8 GHz processer (Intel Core i7; Intel) and 16 GB memory. QSM was normalized relative to the CSF in the posterior horns of the lateral ventricles, avoiding the surrounding brain tissue and choroid plexus.

### Segmentation

Segmentation of dissecting intramural hematomas and atherosclerotic calcifications was done twice independently by two readers (D.H.K. with 7 years of experience and A.A. with 9 years of experience in neuroradiology) layer by layer using a 3D Slicer (https://www.slicer.org) on QSM images. The first and second measurements were done with at least 2-month intervals in both observers.

### Radiomic features extraction

The extraction of radiomic features was done using PyRadiomics package, a comprehensive open-source python package^56^. The first-order features were obtained from histogram analysis of voxel values within the region of interest (ROI) and shape from the morphologic characteristics^35^. The second-order features included gray-level dependence matrix (GLDM), gray-level co-occurrence matrix (GLCM), gray-level run-length matrix (GLRLM), gray-level size zone matrix (GLSZM), and neighboring gray-tone difference matrix (NGTDM). For each ROI, a total of 107 radiomic features were extracted as follows: 18 first-order features, 14 shape features, and 75 texture features (14 GLDM, 24 GLCM, 16 GLRLM, 16 GLSZM, and 5 NGTDM).

### Statistical analysis

The mean and standard deviation of mean and median values of dissecting intramural hematomas and atherosclerotic calcificationswere presented. ICC and wCV were used for intra- and interobserver reproducibility of the mean and median values. The strength of reliability of the ICC was categorized as follows: < 0.20, poor; 0.21–0.40, fair; 0.41–0.60, moderate; 0.61–0.80, good; 0.81–1.00, excellent. To determine the intra- and interobserver reproducibility of the 107 radiomic features obtained from QSM, CCCs as defined by Lin^57^ and overall CCCs as defined by Barnhart et al*.*^58^ and ICC were calculated. Radiomic features with CCC and ICC > 0.85 were regarded as stable. Statistical analysis was performed using MedCalc version 20.014 (MedCalc Software Ltd) and R version 4.1.1 (R Foundation for Statistical Computing). *p* values < 0.05 were regarded as statistically significant.

## Supplementary Information

Supplementary Information.

## Data Availability

The data generated or analyzed during the study are available from the corresponding author by request.
