# Supplementary material for: Feasibility and intra-and interobserver reproducibility of quantitative susceptibility mapping with radiomic features for intracranial dissecting intramural hematomas and atherosclerotic calcifications
Source: Sci Rep. 2023 Mar 4;13:3651. doi: 10.1038/s41598-023-30745-2 (PMC9985647; doi:10.1038/s41598-023-30745-2)
Supplement: Supplementary file 1 — Supplementary Information. [file 41598_2023_30745_MOESM1_ESM.docx]

***Supplemental materials***

*Imaging Protocol*

The imaging parameters for T1WI were as follows: repetition time 600 ms; echo time 17 ms; matrix 320 × 160 × 288; field of view 160 × 80 × 160 mm; number of excitations 1, voxel of 0.5 × 0.5 × 0.5 mm^3^. The acquisition time for each sequence was approximately 7 minutes 21 seconds. The imaging parameters for T2WI were as follows: repetition time 1000 ms; echo time 122 ms; matrix 320 × 160 × 288; field of view 160 × 80 × 160 mm; number of excitations 1, voxel of 0.5 × 0.5 × 0.5 mm^3^. The acquisition time for each sequence was approximately 7 minutes 23 seconds.

*Diagnostic Criteria for Intracranial Arterial Disease*

Atherosclerosis was diagnosed in following criteria: 1. Radiologic findings: Either atherosclerotic stenosis (CTA) or eccentric wall thickening or eccentric contrast enhancement of arteries (VW-MRI); 2. Clinical findings: positive vascular risk factors ((age [men > 50 years, women > 60 years], hypertension, diabetes mellitus, hyperlipidemia, obesity, and smoking); 3. Exclusion criteria: cardioembolism, greater than 50 % stenosis of extracranial arteries proximal to symptomatic intracranial stenosis, non-atherosclerotic vasculopathy such as vasculitis, or Takayasu’s arteritis ^1,2^.

Dissection was classified as definite and suspected case according to SASSY Japan criteria (The Strategies Against Stroke Study for Young Adults in Japan). Intimal flap, double lumen, or suspected criteria with clear geometric change were considered as definite dissection findings. Suspected dissection included pearl and string sign, tapered occlusion (CTA), intramural hematoma, and aneurysmal dilatation (VW-MRI) ^3,4^. The dissecting flap was classified as a layer crossing the arterial lumen extending into the arterial sidewall, and the double lumen was defined as false and true lumen with an intervening intimal flap. The aneurysmal dilatation was observed as an increase in the outer diameter compared with the adjacent normal-appearing arteries, while the dissecting aneurysm was defined as an increase in true and false lumens compared with the adjacent normal-appearing arteries ^5^

1 Kim, Y. J. *et al.* High resolution MRI difference between moyamoya disease and intracranial atherosclerosis. *European journal of neurology : the official journal of the European Federation of Neurological Societies* **20**, 1311-1318, doi:10.1111/ene.12202 (2013).

2 Swartz, R. H. *et al.* Intracranial arterial wall imaging using high-resolution 3-tesla contrast-enhanced MRI. *Neurology* **72**, 627-634, doi:10.1212/01.wnl.0000342470.69739.b3 (2009).

3 Maruyama, H. *et al.* Spontaneous cervicocephalic arterial dissection with headache and neck pain as the only symptom. *J Headache Pain* **13**, 247-253, doi:10.1007/s10194-012-0420-2 (2012).

4 Park, J. E. *et al.* Comparison of 3D magnetic resonance imaging and digital subtraction angiography for intracranial artery stenosis. *European Radiology* **27**, 4737–4746, doi:10.1007/s00330-017-4860-6 (2017).

5 Park, K. J. *et al.* Multi-Contrast High-Resolution Magnetic Resonance Findings of Spontaneous and Unruptured Intracranial Vertebral Artery Dissection: Qualitative and Quantitative Analysis According to Stages. *Cerebrovasc Dis* **42**, 23-31, doi:10.1159/000444315 (2016).

Table 1. The volume and reproducibility of regions of interest

| VOI volume | Dissecting intramural hematoma | |  | Atherosclerotic calcification | |
| --- | --- | --- | --- | --- | --- |
|  | Reader 1 | Reader 2 |  | Reader 1 | Reader 2 |
| Mean values (mm^3^) | ^a^141 ± 127 | ^a^127 ± 114 |  | ^a^59 ± 79 | ^a^48 ± 53 |
| Range (mm^3^) | 3.1–370.9 | |  | 15.5–743.9 | |
| Reproducibility | ICC | |  | ICC | |
| O1 intra | 0.890 (0.669–0.954) | |  | 0.985 (0.967–0.993) | |
| O2 intra | 0.907 (0.803–0.954) | |  | 0.864 (0.683–0.941) | |
| 1st inter | 0.918 (0.844–0.957) | |  | 0.898 (0.779–0.954) | |
| 2nd inter | 0.913 (0.757–0.962) | |  | 0.963 (0.903–0.985) | |
| Intra and inter | 0.901 (0.817–0.948) | |  | 0.942 (0.894–0.972) | |

ICC = intraclass correlation coefficient, O1= observer 1, O2 =observer 2, intra=intraobserver reproducibility, inter=interobserver reproducibility, 1st inter = interobserver reproducibility between the first measurements of observer 1 and 2, 2nd inter = interobserver reproducibility between the second measurements of observer 1 and 2

^*^ Mean values indicate mean±standard deviation.

^**^ Parentheses indicate 95% confidence intervals.

Fig. 1


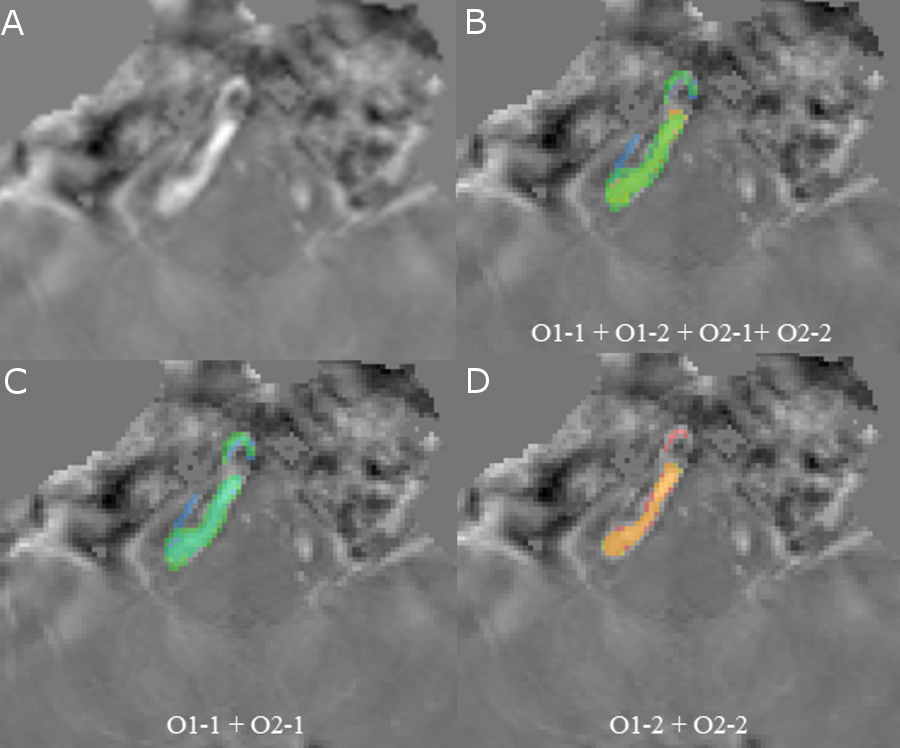


A dissecting intramural hematoma in a 50-year-old male patient. (A) The lesion shows high susceptibility values (mean, 0.328 ppm; median, 0.319 ppm) on quantitative susceptibility mapping (QSM) (arrows), suggesting the paramagnetic nature of the hematoma. (B-D) Overlay of ROIs on QSM images.

Fig.2
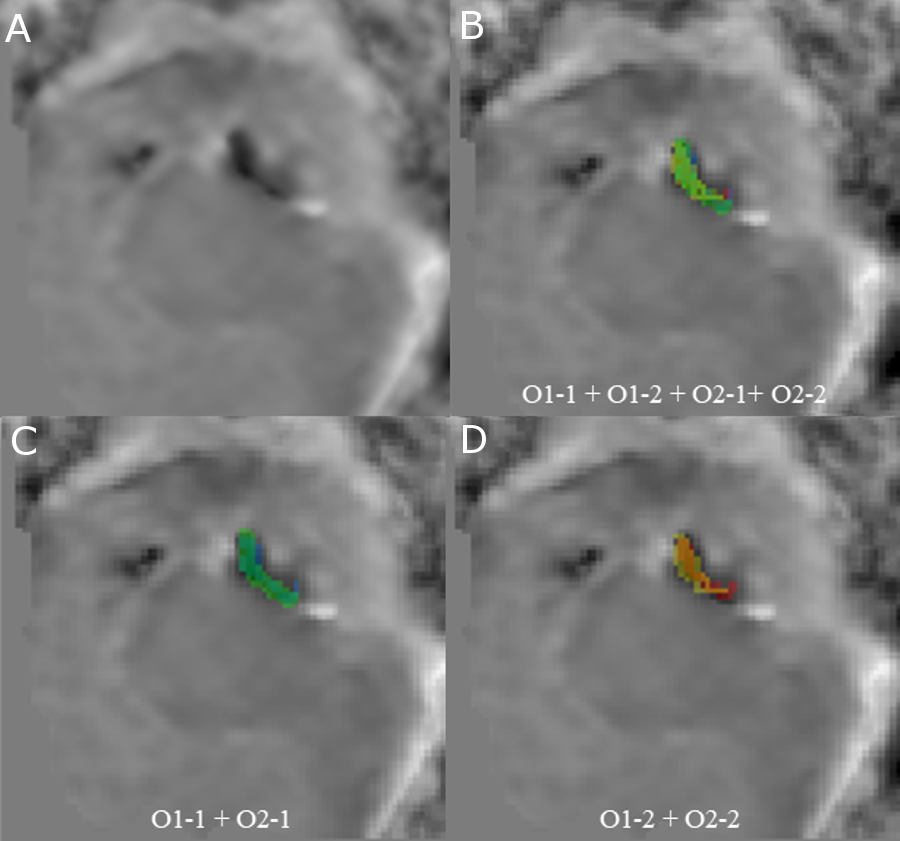


An atherosclerotic calcification in a 68-year-old male patient. (A) The lesion shows low susceptibility values (mean, -0.257 ppm; median, -0.265 ppm) on quantitative susceptibility mapping (QSM) (arrow), suggesting the diamagnetic nature of the calcification. (B-D) Overlay of ROIs on QSM images.
